# The New Conscience

**Published:** 1913-04

**Authors:** 


### The New Conscience.

Verily, this expression has become a reality. A wave of moral reform is sweeping over the entire country, and threatens to be a deluge of cleansing waters, ere its tide recedes. You have but to recall the recent vice commissions which have been appointed from Portland to Boston, that have boldly given expression to the sentiment of the people; State legislative committees now investigating the relation between vice and the low wages of women; individual commissions that have exposed the connection between political graft, and commercialized vice; the establishment of bureaus of social hygiene for the scientific study of the cause and prevention of vice; societies for the specific instruction of sex hygiene and the inclusion in our normal schools instruction on sex hygiene for the training of teachers.

The agencies that are fostering this growing sentiment are the changing attitude toward the economic position of women; the medico-scientific fact that incontinence is not a physiological necessity; the realization of the great toll venereal diseases and vice are making on society, and the recent decision of the Supreme Court, which makes prostitution a crime and gives the State the right to control its moral status.

Regarding segregation, the Portland commission has this to say:

"The history of every restricted district in the country has been the history of police and political corruption, of crime and bloodshed and scandal. Far from eliminating graft, such districts have proved the nursery of official blackmail, the central point from which the scheme of protection has reached out.

"Segregation does not segregate; deals only with a small percentage of the sexually immoral; promotes and justifies professional prostitution; does not reduce clandestine immorality; helps to establish a double standard of morality by stigmatizing the woman and ignoring the moral responsibility of the man; rests on the false presumption that sexual immorality is necessary; fosters the debauchery of the sex instinct; promotes the spread of disease, and affords official absolution for illegal and immoral conduct."

It has been said that when any considerable number of men question the necessity of an evil, it marks the beginning of the end. May this be true of this national problem of commercial vice. And here again the voice of Eugenics speaks. The largest number of public prostitutes are recruited from the class of feeble-minded. Is it not good logic to say stop the breeding of the feeble-minded and you will check the greatest factor in the promotion of vice?

